# Survey on Optical Wireless Communications-Based Services Applied to the Tourism Industry: Potentials and Challenges

**DOI:** 10.3390/s21186282

**Published:** 2021-09-19

**Authors:** Lidia Aguiar-Castillo, Victor Guerra, Julio Rufo, Jose Rabadan, Rafael Perez-Jimenez

**Affiliations:** Institute for Technological Development and Innovation in Communications, Universidad de Las Palmas de Gran Canaria, 35017 Las Palmas, Spain; laguiar@idetic.eu (L.A.-C.); jrufo@idetic.eu (J.R.); jrabadan@idetic.eu (J.R.)

**Keywords:** optical wireless communications, visible light communications, optical camera communications, internet of behavior, smart destination, tourism

## Abstract

In this paper, we explore the potential applications of Optical Wireless Communications in the tourism industry, considering both indoor and outdoor scenarios and different transmission speeds. They range from high-speed atmospheric outdoor links (Free-Space Optics (FSO)) to indoor systems based on high-speed lighting networks (known under the trade name LiFi©) or low-speed services support the Internet of Things networks, using visible light (VLC) or IR emitters, with receivers based on either on classical photodiodes or in image sensors, known as Optical Camera Communications. The avant-garde applications of this technology have been studied focusing on three possible use scenarios: the traveler himself, in what we have called TAN (Tourist Area Network); the tourist facility, which includes not only the hotel but also leisure areas (theme parks, museums, natural protected areas) or services (restaurants, shopping areas, etc.); and the entire destination, which can be both the city or the territory where the tourist is received, within the paradigm of the Smart Tourist Destination (STD). In addition to the classic services based on radio frequency and wired broadband networks, these technologies will make it possible to meet the tourist’s challenging needs, the establishment, and the destination. Besides, they cover the services imposed by the new marketing services related to location or context and feed the big data systems used to study tourist behavior.

## 1. Introduction

Hospitality and tourism have been well recognized as the most vibrant industry driving the service sector in the globalized world. Its substantial contribution to national income and foreign exchange reserves, employment and local business, social infrastructure, international relations, and peace has been acknowledged. It has also been associated with, at least in part, the removal of poverty and inequality in certain places, unless it is usually considered to create only low-qualified jobs. The Manila Declaration on World Tourism of 1980 recognized its importance as “an activity essential to the life of nations because of its direct effects on the social, cultural, educational, and economic sectors of national societies, and their international relations” [1]. The emergence of the COVID-19 pandemic in 2020 has marked the end of a decade of continuous growth in the tourism sector, which led it to be one of the largest sources of income for many countries throughout the world. The combined value of this market reached 1.5 trillion dollars in 2018, around 3.6% of world GDP, and 30% of the services market, in addition to 10% of global employment. Tourism is the third largest export sector in the world economy after chemicals and fuels [2]. This industry includes not only travel for pleasure or business, but also the marketing, management, and entertainment techniques aimed at the tourist; the areas of transport and hospitality; and the study of the effects that the tourist activity has on the city or territory where it takes place (tourist destination). Increasingly, this activity is dedicated to specific market niches, such as ecological (or green) tourism; health tourism; cruise or rail travel; stays in specific rural, agricultural, or tribal areas; sports; or adventure. Furthermore, there are specific travel routes dedicated to those who are passionate about cinema, collecting, culture, or heritage, or simply professional meetings, business incentive trips or attendance to conferences or exhibitions. The latter are generally grouped under the name MICE (meetings, incentives, conferences, and exhibitions) [3]. Traveling is also a way of opening our minds and increase our knowledge about other cultures, gastronomy, or artistic heritage. As St. Augustine stated, “The world is a book and those who do not travel only read one page.”

The pandemic and the almost total confinement imposed in response to its expansion have led to the sector’s roughly total paralysis, so the latest edition of the UNWTO Barometer shows drops of up to 98% compared to 2019. Concerns regarding safety issues associated with travel, the reappearance of the virus, the risks of new confinements or curfews, the lack of reliable information, and the deterioration of the economic environment appear as factors that also undermine consumer confidence. However, vaccine arrival in 2020 allows us to hope for a recovery in the second half of 2021, and more surely in 2022 [4]. Every crisis involves a reflection on how to approach the day after the pandemic. Many tour operators are focusing their efforts on the increase of competitiveness by reducing costs and increasing the quality of the offer, or by strengthening the segmentation of the offer to cover new specific niches. In all cases, the use of information and communication technologies is seen as the basic tool to incorporate solutions already present in other sectors such as logistics, security, transport, education, or health, all of them also present as integral parts of the tourism industry.

Another factor is that, in their places of origin, travelers already enjoy a wide range of digital services, an offer that is expected to be at least similar in the destination. This implies the availability of communication networks that guarantee ubiquitous access to data and value-added services, such as secure economic transactions, real-time location systems, assistance and information to travelers in heritage or cultural places, or the provision of remote medical assistance. It makes the need for new technological services in tourist sites at least similar, if not superior, to those of other cities and facilities. The translation of smartcity concepts to this industry is reflected in the concept of “smart tourist destination” [5], which includes not only the availability of high-speed data networks, but also guide services and the safe use of cultural or patrimonial facilities. It also comprises compliance with the sustainable development goals (SDG), as support for sustainability policies is a fundamental part of the destination image in the face of an increasingly environmentally conscious tourism. This is reflected in systems for the wastewater control, the presence of pollution or pollens, or the monitoring and recycling of garbage. Traffic control policies are also introduced to reduce the impact of traffic jams, parking problems, and the possibility of accidents, especially when the floating population greatly exceeds the resident population. For this reason, the introduction of intelligent driving services based on vehicle-to-vehicle (V2V) or vehicle-to-infrastructure (V2I) communications is also an issue of great interest. The availability of advanced public electronic government services is also desirable to facilitate the completion of procedures for travelers in case of illness or loss of documentation. In some cities, specific services are also proposed for people with special needs such as children, elderly, or people with health disorders [6]. This new vision of tourism as a digital industry also increases the need for highly qualified jobs in these destinations, helping the creation of technology-based companies and specific university research groups on these subjects.

This paper presents a study on the possible applications of wireless optical communication technologies in the tourist environment, identifying possible feasible niches and their implementation challenges. This includes the ultraviolet, infrared, and visible bands (350 to approximately 1000 nm), which offer a wide unregulated bandwidth, compatible with radio-frequency systems and available for these new services. This follows previous work by this research group [7,8], where a survey was presented with a panel of experts to discover advantages, challenges, possible disadvantages, or contributions to the image of the tourist destination or facility and its potential advantages [9]. It is organized as follows. First, the needs of information and communications technologies for the smart tourism ecosystem is are presented, divided into three application layers: Smart destinations, tourism smart facilities, and services to the individual tourist. Then, a description of the available OWC solutions and their technical capabilities, even compared to classical RF, is made in Section 3. They are then analyzed in the context of the application scenarios’ technological needs in Section 4, Section 5 and Section 6. Section 7 presents some ideas for the post-COVID tourism industry, and finally, some conclusions and future work to be developed in this area are shown.

## 2. ICT and Smart Tourism: Trends and Scenarios

**Figure 1:**
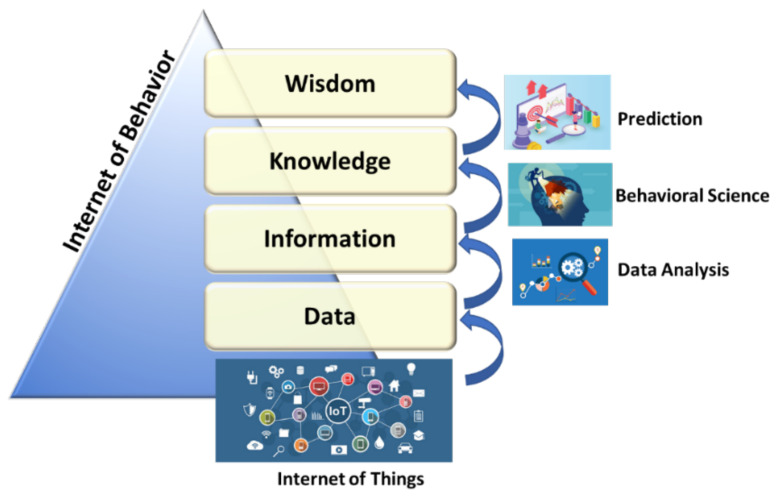
Scheme of internet of behavior from data extraction.

Unless 2020 has been a litmus test for the Information Technology sector, nothing can be compared to its effect on the tourism sector. Not even the significant war conflicts of the last century can be compared to the global pandemic phenomenon: social distancing, confinement, or the indefinite suspension of activities such as tourism or travel have been factors of disruption in tourism, transport, hospitality, or the destinations themselves. The pandemic has affected a sector that was suffering a deep transformation due to the impact of ICT, with job destruction due to process automation, and new opportunities arising around technologies. Artificial Intelligence, 5G, or the Internet of Behavior (IoB; see Figure 1) [10], used to digitally link a person to their actions, e.g., linking an image as documented by facial recognition with an activity such as purchasing a plane ticket can be tracked digitally. Some of the trends on ICT on tourism can be summarized as follows.

- Robotic process automation of repetitive and systematic tasks. In the tourism sector, they are prevailing in cleaning spaces and the check-in and check-out processes [11]. In these scenarios, also AI engineering is being used [12].
- Cybersecurity, as many of the implemented systems are improvised by companies (especially in small hotels), security gaps have led to the emergence of opportunists launching ransomware, botnet, and fishing threats [13].
- Cloud solutions and edge computing. The “as a service” model allows employees to use work tools previously carried out in the cloud. It is also of great help for e-commerce processes without the need to invest in infrastructures (restaurants, for example, have given their gastronomic proposals to delivery platforms at home). These cloud solutions are essential to maintain the service when limitations mobility and schedule [14]. Furthermore, the “operations anywhere” paradigm allows services to be available on any device, computers, tablets, and mobiles, making workers able to carry out their activity from anywhere [15].
- 5G is foreseen for telecommunications services and remote control of infrastructures or collaborative works that take this technology’s essential support. Furthermore, it opens the door for IoB, based on personal data (biometrics, sensors, and daily activity routines), to offer personalized and tailored services.
- Sustainability as a parameter for modeling services and products, and considered a relevant variable for validating projects [16].
- Blockchain-based systems, e.g., for identification, easing the check-in at hotels and airports, streamline processes, and provide more control and privacy over tourist data [17].
- The pandemic’s reality makes these technologies arise as an aid to face new challenges, hitherto inconceivable. Any technology connected with contactless tourism, capacity management, and social distance maintenance will be rewarded with the interest and follow-up of the agents involved [18].

**Figure 2:**
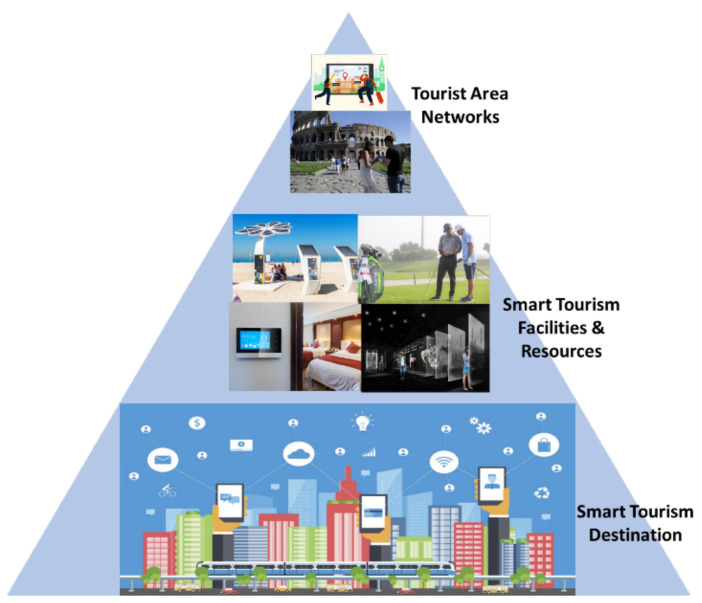
Application scenarios for ICT in the tourist industry.

The massive amount of data generated from these technologies provides knowledge that can generate new and better services, making destinations qualified as “smart” and attractive through their analysis. The use of these technologies for smart facilities and destinations allows detecting behavior patterns, helping practitioners and policy-makers in efficient and rapid decision-making. The SoCoMo/SoloMo (Social-Context/Location-Mobile) marketing paradigms [19] frame the use of context, social media, and mobile phones, and define how local service providers and destinations can extract information from tourists and know their behaviors, tastes, and movements. Their information inputs are divided into external and internal contextual information, with areas ranging from smart destinations, smart tourist facilities, and even tourism services. In this work, three technological scenarios are presented (see Figure 2): technologies for the tourist itself (we can define them as Tourist Area Networks (TANs)), for the tourist resource (hotel, restaurant, theme park, port or airport, cruise ship, museum, or heritage area), and the tourist destination, understood as the whole of a city or a specific territory (an island, a national park). Some current initiatives are presented in Table 1:

### 2.1. Smart Destinations and Tourist-Centric Services

Even before the COVID-19 pandemic, the tourism sector was immersed in an unprecedented change driven by new technologies and the appearance of new actors and disruptive business models in the digital context. The nature and scale of these new challenges on the tourist destination require redefining tourism policies and developing new planning and tourism management tools. Some technology trends in smart destinations, according to industry experts, are the use of destination-level platforms integrating data from the three previously mentioned sectors (tourists, tourism facilities, and destination (comprehensive destination planning)) [20]. The extensive use of applications based on Business Intelligence, Big Data, or Smartphone apps allows the generation of management tools for tourist companies, helping in their digital transformation. Moreover, it contributes to citizens and visitors promoting the destinations, while providing real-time information about their habits, supporting public and private managers [21]. The sensor-based monitoring of tourist areas, destinations, or infrastructures allows their full integration in management platforms [22], while promoting sustainability and good environmental management practices [23]. Virtual and augmented reality (VR/AR) increases the perceived value of the tourist service [24]. All of them are based on the deployment of a complete 5G coverage, and the implementation of access points and Wi-Fi connection [25]. In the frame of the current pandemic crisis, as additional solutions cameras have been added (video surveillance or even located on drones) for measuring the number of visitors, Bluetooth-based presence sensors and beacons to control people flows, wristbands or mobile apps for distance control, and artificial intelligence algorithms to ensure the maintenance of social distance and reduce crowds and queues.

Besides, digital technologies will stimulate tourism destinations to improve connectivity on their facilities and transport services to safely cope with the exponential use of data required in the sector. This fact can undoubtedly become the economy of tourism data, without forgetting the technologies’ contribution to the virtual and contactless relationship between professionals in the sector and tourists and the security and advantages that teleworking can provide to these practitioners.

Finally, the benefits offered by the technologies for the follow-up of COVID-19 cases and contact tracing (by geolocation or Bluetooth, depending on the legislation of each State allows) or the use of thermographic cameras for taking the temperatures of employees and tourists must also be considered. Other types of applications could be robots for cleaning and disinfection tasks (for example, equipped with ultraviolet light), digital control systems for sanitary measures using QR codes, and disinfection machines (full-body systems for passengers in airports).

### 2.2. Tourism Smart Facilities

Many ICT-based services are being incorporated into the hospitality facilities: self-service technologies for check-in/out, front-office procedures supported by chatbots, room access through smartphones, wearable devices or biometric technologies, smart tags on luggage, voice activation systems for devices, lighting systems with motion detection, digital charts/menus, applications with real-time interaction with tourists, or advanced digital identity systems. These solutions also contribute to keeping contacts to a minimum, so their use has been promoted during COVID time. Personal mobile devices for many of these tasks reduce risks and create security for tourists and professionals in tourism companies, making clear that the future of tourism will be digital and mobile [26]. In the medium/long-term, this crisis represents an opportunity to advance in the digital transformation process, which had already begun and presents different degrees of progress depending on the subsectors. Outstanding entities such as the World Tourism Organization [27], the European Commission [28], Exceltur [29], or SITA (the global association of technology applied to air transport) [30] base an essential part of the future recovery of the sector on digitization. The key elements they propose are listed below.

- Digitization of the complete value chain of the sector, including contact, customer service, and the companies’ internal processes, and improving the digital identity of tourist brands.
- Data analytics and market intelligence.
- Increase in cybersecurity throughout the value chain.
- Improvement of the tourist experience by applying emerging technologies, such as 5G, to make it more digital and immersive on the road to smart tourist destinations.
- Boost innovation and entrepreneurship in the sector, emphasizing talent management and staff training in digital skills and increasing teleworking in areas where feasible.

These elements should help create a future scenario based on the return of confidence and the sector’s recovery and momentum within digital, mobile, and intelligent tourism.

Regarding tourist facilities, interest is focused on the use of location systems, both for guiding and for monitoring people, especially those who are most sensitive for security reasons or those with special needs. An example would be a leisure theme park or leisure tourism facility [31], where safe areas could be defined where children or the elderly could function without requiring assistance, also providing rest periods for parents or caregivers. The monitoring of tourist activities is a key factor in modern Customer Relationship Management (CRM) systems, especially those based on SoCoMo (Social Context Mobile) paradigms, which is based on contextual information provided by a wide range of sensors in a smart destination, which provide information in real time to reflect the tourist experience. This information comes from a wide variety of connected devices (where VLC/OCC systems could potentially be included) and from the monitoring of the daily activity of tourists, for example, through IoT devices and from their contributions on social networks and other media. All these inputs are processed as a source of big data to provide tourism stakeholders with valuable information about the behavior and preferences of each segment of travelers and their responses to commercial stimuli, in what is now known as the Internet of Behavior (IoB). These applications are especially suitable for the orientation and management of information in museums [32,33], commercial areas [34], and cultural facilities [35], where the directionality of the optical links and their intrinsically cellular character, being confined by the walls, becomes an advantage, e.g., we can discriminate what specific picture is being contemplated or what type of products attract a tourist to a store. Some experimental experiences have already been verified in the use of VLC systems to allow automatic hotel check-in, where the cellphone screen acts as a “key” for the verified hotel room access system [36]. This reduces the check-in time (and the cost) for both the tourist and the hotel. The use of graphic panels has also been proposed as sources of information for OCC systems (for example, in airports or commercial areas), and as secure wireless transmission systems. The combined use of OCC and VLC systems can be also included in the models of IoT networks in tourist facilities [37]. These networks, which would work in conjunction with RF systems or alone depending on whether or not there are electromagnetic compatibility requirements, simply expand the available bandwidth.

Nevertheless, as the interest in new solutions and digitalization evolves, some challenges are to be faced by tourism companies (in particular, SMEs) for seizing these new opportunities, as could be inadequate access to digital infrastructure, insufficient resources, or (especially in the COVID and post-COVID times), the necessity on focusing on business survival, making them risk-averse to innovation. On the other hand, tourism success contributes to local economies. Furthermore, sustainable tourism should not be considered a particular form of tourism, but all tourism forms should strive to be more sustainable.

## 3. OWC Technologies Suitable for the Tourism Industry

**Figure 3:**
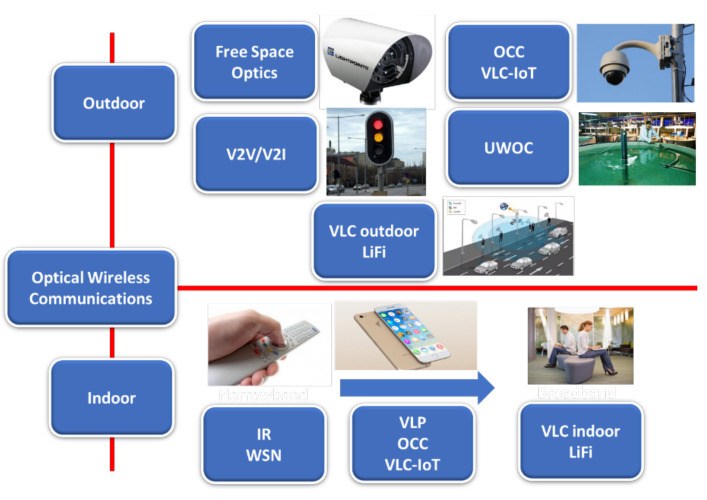
OWC technologies to be used in the tourism industry.

Under the name of Optical Wireless Communications is grouped a set of technologies shown in Figure 3. They comprise systems based on different wavelengths and with different characteristics [9,38,39,40]. FSO (Free Space Optics) links are point-to-point links that use laser connections, usually in the 1550 nm band, and allow covering spans that usually reach up to 4 km. Another set of systems is based on non-coherent, wide-area sources, either in visible wavelengths (and which are grouped under the name of Visible light Communications (VLC) systems), infrared (IR), or ultraviolet (UV). In VLC and IR systems, two basic detection technologies can be used photodiodes (PD) or image sensors (cameras). In this last case, we talk of Optical Camera Communications (OCC). We can group applications based on the application environment (Figure 3).

FSO is a simple and cheap alternative to provide broadband data access to relatively remote areas without the need for a wired infrastructure. This is a common problem in tourist facilities lacking of 5G/LTE coverage and spread over large remote areas such as resorts in natural parks, beaches, or golf courses. It is based on a modulated, line-of-sight laser transmission through the atmosphere (or vacuum in communications between satellites), using infrared radiation, usually in the 1550 nm band. It offers several advantages over microwave links such as the lack of spectrum regulation, security against intrusions, or requiring a less strict criterion in terms of clearance areas, such as the Fresnel zone [41,42]. This is the area around the line of sight that must be clear of obstacles to avoid degradation in signal quality [43], and its size is proportional to the wavelength of the signal, so it is smaller in an FSO system than a conventional radio link.

An FSO communication system consists of three main parts: transmitter, free space channel, and receiver. Transmission can be done with a direct or externally modulated beam [44]. Generally, a vertical cavity surface emitting laser (VCSEL) is used, as they are cheaper and have better performance than the FP and DFB ones [45]. Transmission takes place through the atmosphere, and is therefore subject to the action of phenomena such as scintillation, scattering, absorption, and atmospheric turbulence that affect the performance of the link [46]. The optical receiver uses a collecting telescope that is used to focus the incoming optical light from the channel onto the photodetector (PD). This allows multiple uncorrelated beams to be received and subsequently averaged and focused to the photodiode [47].

**Figure 4:**
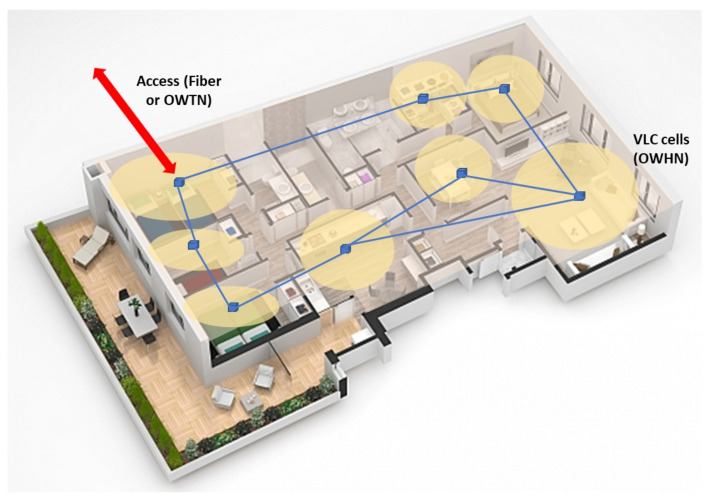
Optical wireless data structure.

FSO systems can be classified in two main categories: single- and multibeam. A single-beam system is easier to align, simpler, and cheaper, but more sensitive to atmospheric turbulence or fog, which increases the probability of error under those conditions. In the other hand, the combination of a multibeam FSO system reduces the effect of beam spread [48], and the degradation of the signal in turbulent conditions, ensuring better availability of links in coastal regions—as many tourism areas—where low visibility due to morning fog is common [49]. To increase link capacity, each of these links can support DWDM wavelength multiplexing techniques [50]. From a functional point of view, FSO networks can be classified into three main types: (i) Optical Wireless Satellite Networks (OWSN), (ii) Optical Terrestrial Wireless Networks (OWTN), and (iii) Optical Wireless Home Networks (OWHN). These last categories can be integrated as subnets that operate, as shown in Figure 4, and are the more suitable to be employed in tourism facilities. OWTNs are used as a backbone in broadband Internet access networks, while OWHNs can be used in offices, shopping malls, hotels, or heritage buildings to give the user access by creating a LAN with a cellular structure, where each cell is one of the divided spaces of the building. Typically, each cell has a base station connected to multiple terminals with short-range WiFi or optical wireless connections. These optical connections are usually based on VLC systems.

VLC was conceived as a complementary technology to short-range RF systems, as this spectrum is becoming progressively saturated, VLC is based on a part of the spectrum that is not regulated, allowing VLC to have a bandwidth capable of transmitting very high data rates without a license. They are also safer against unwanted access to data and are not subject to the increasing susceptibility of RF emissions, considering that they can be harmful to humans if their intensity exceeds a certain threshold [51]. Furthermore, the luminaires used for VLC systems are energy-efficient despite their high-light intensity, and they are also very low cost. In [52], a comparison between the performance of VLC and RF technologies can be found. The main advantage of VLC systems is that they do not require a strict pointing as FSO, as VLC can even work with signal reflected off walls or obstacles. On the other hand, as it is based on a non-coherent detection technique, the maximum achievable data rate dramatically decreases as the link distance increases.

The use of VLC systems in vehicular communications is still under development, but it is the object of great scientific and commercial interest as it will allow acquiring information in real-time to keep drivers and pedestrians safer. In addition to communication technologies based on radio-frequency (RF), VLC emerges as a complementary solution due to its simple design and low cost of deployment. V2V communications using headlights or taillights seem straightforward to be implemented for short distances, allowing applications such as platooning or convoy navigation [53,54]. However, the use of VLC applications in V2I communications faces challenges derived from vehicle mobility [55]. The limited coverage range of the access points and the high speed of the vehicles only allow you to create links for a limited time. Consequently, conventional handover schemes for VLC networks are not applicable in these scenarios, and it is necessary to create a network topology and a communication model between nodes to facilitate fast handover between cells [56,57].

These are based on main types of LEDs acting as VLC sources: Phosphor LED (PC-LED) and multi-chip LED (RGB LED). Reception is typically accomplished using photodetectors (PD) that act as a quadratic law optoelectronic transducer sensor, which detects and responds to light radiation striking its surface. Unlike image sensors, individual PDs offer speeds in the 100 Mbps range if they meet certain requirements such as sufficient bandwidth, very low noise, and very high sensitivity in the wavelength range in which it operates. In addition, the effects of temperature variations of the PDs must be minimal and their MTTF (Mean Time Between Failures) must be very long. Commercial PD PINs and APDs meet all those requirements, working at different wavelengths that are determined by their chemical composition.

In VLC, the modulated signals are carried by the optical intensity of the illumination source through different modulation schemes that must provide real and positive values of optical intensity [58], which generally invalidates the use of amplitude and phase modulation techniques. Another problem is fluctuations in illuminance levels (flickering), which are a nuisance when used in the presence of humans, limiting the minimum frequencies to around 200 Hz [59]. The most widespread modulations are On–Off Keying (OOK), Pulse Position Modulation (PPM), Color Space Modulations, and Optical OFDM [60], which are used in conjunction with Run-Length Limited (RLL) codes to prevent these flicker effects. Error correction can be executed by adopting Forward Error Correction (FEC) techniques such as Reed Solomon (RS) [52].

Another problem to be solved is the management of multiple access as it is common for luminaires with multiple sources to be used to achieve adequate levels of illuminance. This means having several LEDs per source that can be used as transmitters, driving to a MIMO-VLC model [61], which means an improvement in spectral efficiency by establishing a parallel communication that also requires several detectors. The use of MIMO schemes allows in turn to relax the alignment requirements, easing their functioning in mobile wireless systems [62]. When comparing those systems with RF-MIMO, the design and implementation of optical MIMO systems for VLC is more complex due to the limited spatial diversity of these small wavelengths signals. Diversity receiver design is then focused more on signal combination techniques, instead of considering the signals captured in the different receivers as independent [63,64].

A natural extension of optical MIMO systems is the use of a matrix of photodetectors such as those that form an imaging camera, as receivers. This has given rise to OCC (Optical Camera Communications) systems. OCC (also known as Image Sensor Communications [65]) is a communication technology that uses optical image sensors as receivers, based on the IR or visible spectrum. Compared to other types of OWC technologies, OCC relies on commercial cameras, such as those used in smartphones, drones, vehicles, or surveillance systems, that can be used as receivers with almost no modification. OCC receivers are based on arrays of up to millions of pixels, providing a high degree of freedom to transmit data and manage access for multiple users. In addition, current image sensors allow transmission over the color domain. All these advantages in cost, popularity, and information transmission capacity have opened numerous fields of potential interest such as IoT, location, motion capture, and intelligent transport systems (ITS). On the other side, it is limited to low data rate services and is affected by blur effect, unstable frame rate, and random image losses (mainly due to the processing time in mobile phones).

**Figure 5:**
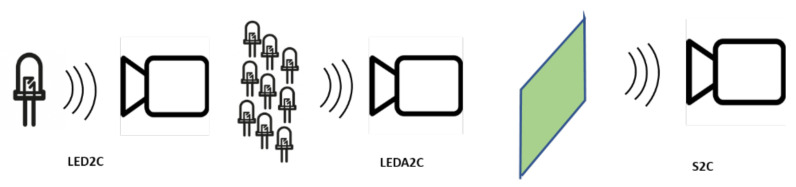
OCC categories.

According to the type of emitters, OCC can be divided into three categories of increasing complexity: a single transmitting LED and a camera as a receiver (LED2C); a LED matrix as a transmitter and a camera as a receiver (LEDA2C); or a screen as a transmitter and a camera as a receiver (S2C) [66] such as a television screen, a commercial, or traffic information panel. The light reaches the receiver lens and then passes through the shutter, which controls exposure and can be global (GS) or roller shutter (RS). The difference between them relies in the exposed area, as the global one controls all the image sensors simultaneously, while the roll-up gives access to the image sensors row by row. The captured image contains information as it has been modulated by means of a modification of any of its parameters. Then, using image processing techniques and data extraction schemes, the information carried by modulation (in space, time, intensity, or color of light) can be decoded. Figure 5 depicts the different types of OCC systems.

These low data rate systems make it possible to cover the applications that are included under the name of Internet of Things (IoT), in this case also known as LIoT (Light IoT) [67]. LIoT networks can work in conjunction with conventional networks such as NarrowBand©, IoT, LoRA©, or Sigfox© [68] to work in areas where there may be limitations to the use of RF networks, due to regulatory reasons, or to the presence of interferences or ill propagation (such as hospitals, underground mines or industrial areas) [69,70]. Furthermore, given that the visible optical signal (in the blue-green band) propagates better underwater than the RF systems and with emitters of smaller dimensions and consumption, it makes this solution more suitable for sensor networks or underwater point-to-point communications (UWOC).

There are several communication standards covering the use of wireless optical systems. The first works on VLC standardization came from the Japanese Association of Technology Industries (JEITA), and these standards were later accepted as JIETA CP-1221 and JIETA CP-1222 [71] in 2011, IEEE proposed the VLC standard called IEEE 802.15.7-2011 [72], which has now been superseded by IEEE 802.15.7-2018 to cover a wider spectrum from 190 nm to 10 μm (IR to UV) for short-range OWC [73], and specially for OCC applications. OCC regulation was based on the work developed by a task group working on efficient modulation schemes for Global or Rolling Shutter configurations, system architecture, or the specification of the MAC layers. Higher baud rates are covered by the IEEE 802.15.13 (formerly under Li-Fi proposal [74]), while the International Telecommunication Union (ITU) has also developed and approved its VLC G.9991 standard in 2019 [75], covering three aspects for a high-speed indoor OWC transceiver based on the use of visible light: architecture and Physical and Data Link layers. These standardization activities are broadening the path to new applications and commercial development through companies.

These new specifications can extend the global market for VLC-based applications in critical areas that have already been highlighted as the most important for the tourism sector: location, for commercial or assistance purposes, monitoring via IoT networks, sustainability management, methods payment systems, as well as others under development such as the use of OLED-based portable PAN devices, included in sportswear or commercial video-based steganography to acquire data from information panels or commercial television [76].

In general, OWC systems are designed to work complementary with RF systems, either at a functional level, as both can work in parallel, or combining them, as a radio uplink can be created for a high-speed VLC downlink [77], or an OCC system can be used to carry out handover between cells or RF networks [78,79]. A combined system can also be made, even from the construction point of view, since RF/FSO radio links can be combined in single support. As a result, the atmospheric limitations of each of the technologies can be avoided (OWC are mainly affected by the presence of fog or dust, while is not affected by rain, different to RF that suffers severe fading on rain conditions), or used in combination when weather conditions allow doing that [80,81].

OWC systems present both advantages and disadvantages over other wireless systems: first, they are not limited by regulatory aspects regarding the use of spectrum, and their level of security against undesired data access is much higher than in conventional RF systems. They can also generate bandwidths that are at least similar to, and probably higher than, conventional systems. OWC is a safe technology for the user and is sustainable as it is a dual-use system and is based on low-cost devices and electrical consumption. On the other hand, the commercial implementation of these devices still faces problems due to mobility and shadowing, and the optical signal’s attenuation when undergoing electro-optical conversion processes. Therefore, the different OWC systems can cover specific areas of the communication needs in a tourist facility. FSO systems can work as a distribution backbone from an access point to the global network or connecting separate facilities. Classical VLC links can complement the network services offered by RF systems, both in LAN and other networks such as positioning or access control. Finally, OCC systems are a complement to systems such as Bluetooth to solve personal access network problems, using smartphones as receivers, working with wearable systems or allowing personalized access to information panels. Those aspects will be studied in the following sections.

## 4. OWC on the Smart Tourism Destination

As mentioned in Section 2, the different OWC applications of interest in tourist environments will be presented at three levels of definition: systems and solutions for tourist destinations; for hospitality, leisure, commercial, and recreational areas; and, finally, those dedicated to the traveler, which are grouped under the name of Tourist Area Networks (TAN). The Smart Tourism Destinations (STD) concept emerges from the development of Smart Cities. With technology being embedded in all organizations and entities, destinations will exploit synergies between ubiquitous sensing technology and their social components to enrich tourist experiences [82]. OWC is one of the sets of technologies to be employed to be considered as a support for the STD services, and to obtain data for feeding the big data services used for CRM (Customer relationship management) marketing services.

### 4.1. 5G/6G-Based Services

The fifth generation of wireless communication technologies and standards (5G) offers a higher data rate (up to 10 Gbps, 10 to 100 times better than 4G and 4.5G networks) with a latency of less than 1 ms and with up to 100 devices more connected (and with less battery consumption) per unit of the area compared to 4G networks. A user’s perception of the different communications networks will be that voice and text are available in 1G and 2G. In 3G and 4G, pictures and video become commonplace. In 5G, live ultra-high-definition three-dimensional data can be employed, while in 6G, it is expected that we could have a ubiquitous virtual existence. 5G implementation, unlike previous ones, does not come so much from the need to use the mobile but from the demand of streaming services, IoT networks, or V2V/V2I systems for autonomous driving. According to an Ericsson report [83], by 2024, 5G will reach 45% population coverage, making it the fastest-growing generation in the world.

The full implementation of the 5G network will contribute to generalize the use of VR (virtual reality) applications, 360º video or augmented reality (AR), IoT, and artificial intelligence, which will allow the configuration of the future “immersive tourism” (allowing the user to amplify their participation and improve their interaction with a product, service or tourist destination). This network will also allow the simultaneous connection of more devices (mobile phones, equipment sensors, surveillance cameras, emergency services, and smart streetlights), facilitating the management of a tourist destination integrally and its scalability between high or low season, or in specific situations with a large influx of visitors. At the same time, 5G will favor cloud storage and processing, making possible access to virtual reality contents in more and more destinations, as the cloud VR market is forecast to reach 292 Billion USD in 2025 [84].

To achieve these goals, 5G faces serious challenges where OWC solutions can be integrated. One of the most promising approaches is to embed large numbers of cooperating small cells into the macro-cell coverage area. Alternatively, optical wireless-based technologies can be adopted as an alternative physical layer offering higher data rates [85,86]. Other authors have focused on the new paradigm of human-centric networks with heterogeneous networks using hybrid RF/optical solutions [87,88] or on the problems associated with the management of streaming contents [89].

**Figure 6:**
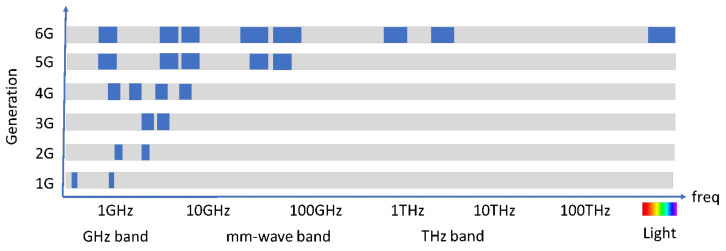
Spectrum usage throughout the past, present, and future mobile generations [59].

**Figure 7:**
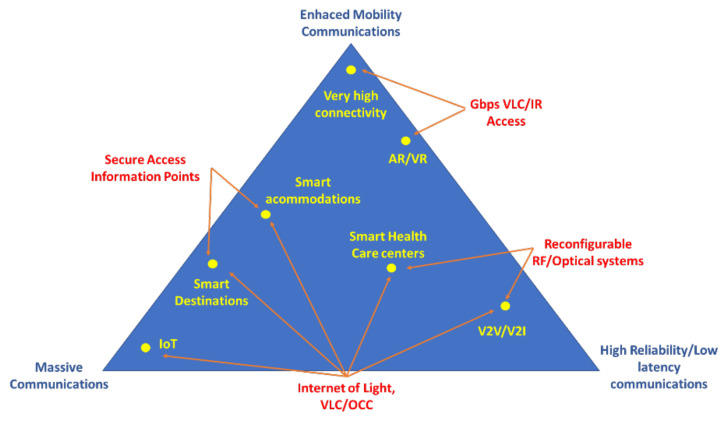
6G VLC-based applications for the tourism industry.

While 5G is being deployed, the first 6G is being designed [90], where promising concepts and technologies are identified and evaluated. As Katz has presented [35], 6G is intended to integrate cellular, short-range, and satellite communications. This new paradigm will integrate different technologies in a heterogeneous network [91,92,93]. Figure 6 shows the spectrum bands to be prospectively occupied by the new standard. As mm-wave and THz operation components will be expensive, visible light communication is a highly qualified technology to be used in 6G as optical components have been widely available for decades, and they are typically low cost [94]. In general terms, propagation in optical wireless communications is quite like that in THz bands, with very short reach and signals being easily blocked by objects; thus, many of the proposed THz applications can be supported by VLC as well [95]. The flexibility, scalability, and relative simplicity of radio can indeed be combined with the inherent security, safety, and privacy of VLC to create a high-performance and robust hybrid communication system [96]. Figure 7 describes some of the uses that the future hybrid VLC-6G network will probably have in the field of tourism.

### 4.2. Building Interconnection and Outdoor IoT Services

A widespread problem in hotels or tourist establishments covering extensive areas (resorts, golf courses, natural parks, or even urban commercial areas) is the interconnection between buildings, as they can be located in protected natural areas or historical city centers. There are limitations to lay cables in these places or areas with relatively low network coverage (as is the case of those installed in remote areas or in developing countries). Such facilities may have a single network point (a fiber or satellite access point) that must be distributed to the entire facility. Ad hoc solutions as NarrowBand IoT©, LoRa©, SigFox©, or legacy systems such as WiMax© do not provide enough broadband, while Bluetooth© or WiFi© extenders are limited in range and do not provide protection against interferences or hacking [97]. One solution for interconnecting long-range access points is using FSO systems [98], by themselves or jointly to RF links [99], as they use unregulated spectrum, do not need a fixed architecture, and the network topology can be easily changed when new access points are needed.

**Figure 8:**
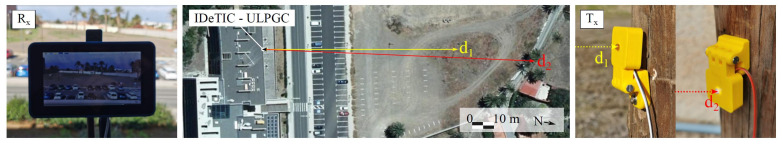
IDeTIC experimental.

Providing solutions to low-speed links, such as supporting internet of things networks, is critical in managing tourist destinations if they are to be configured as smart ones. For this, different technical solutions can be proposed, such as OCC systems. Thus, cameras already installed for traffic surveillance or security could be used, unless, in this case, they would have to work in Global Shutter (GS) format, although it would limit the transmission speed. On the other hand, it simplifies its processing. Another issue would be privacy issues related to captured images, although these images do not need to be stored once processed to extract the information. One possible solution comes from sub-pixel links, where an outdoor GS-based link where communication happens at a sub-pixel level (see Figure 8). The term sub-pixel refers to the fact that the source’s projection area is smaller than the one-pixel area. Recent works [100] show that the LED’S energy affects the adjacent pixels so that the signal can be successfully recovered. These schemes should be combined with link-level solutions to provide a full sensor network topology [101,102,103].

## 5. Smart Services on Tourism Facilities

The second level of application includes systems designed to provide service in accommodation facilities (hotels, apartments, and resorts) or specific resources, such as commercial areas, museums, and sports or leisure areas, such as golf courses, natural parks, or beaches. Therefore, they are areas of strongly variable extension and objectives, and where pre-existing commercial solutions can be applied in other areas such as offices, customer service, or environmental management systems.

### 5.1. Location and Tracking: Health and Leisure Facilities

Location and tracking routines have a large number of applications within the tourism industry. In addition to the traditional sensor location routines typical of IoT systems, which can also be used in tourist establishments in general, travelers demand RTLS (Real-Time Location Systems) services to allow them to find their way around a large resort, a natural area, museum, golf course, or open shopping area [104]. Furthermore, knowing a user’s position and responses when he/she receives a commercial stimulus (for example, an offer of a leisure activity or the time spent observing a particular work of art) allows sectorizing the market and creating personalized marketing offers. This fact also makes it possible to assess results when introducing other resources, such as gamification systems, to stimulate certain activities. RTLS services are already present in many commercial applications, but the tourist entrepreneur does not have access to their specific answers, so it is interesting to carry out specific applications of an establishment, commercial area, or destination. Bluetooth, GPS, and beacon technologies have all opened up new possibilities in delivering location-based information. These services can be used by companies in the hotel and hospitality industry to send messages to customers at the precise time they are most relevant to the recipient. This point may mean, for example, sending SMS messages about menu items at the restaurant when guests are close by, or advertising gym services when they are near the gym. It may also mean sending up-to-date information about local transport links or nearby attractions. Businesses can also use location data to optimize staffing levels. It also makes it possible to study the routes followed by tourists in a given area and minimize the number of people who meet at a given time and place, reducing the sensation of overcrowding of a given attraction. These activities are usually analyzed through big data and are focused on the Social Context Mobile (SoCoMo) paradigm proposed by Buhalis and others [19,105] as a new strategy for improving CRM strategies and systems.

Beyond marketing, location and monitoring routines are essential in welfare or family tourism centers as they allow the delimitation of safety zones for people who need special assistance. Imagine a person affected by a disease that affects his/her memory or spatial capacity and travels with assistance personnel to a hotel or specialized residence. The possibility of having location services allows the affected person (and their carers) more freedom, as they know their position, can track their movements, and can be sure that they are not in danger of leaving the premises or accidentally accessing potentially dangerous areas (swimming pool, kitchens, warehouses, etc.). In the case of family trips with children, specific areas can be set up for games without adults’ presence but monitored by their parents or caregivers, giving them the possibility of enjoying other entertainment at the hotel. Of course, all these location/tracking services should be provided under strict privacy and ethical considerations.

From a technical perspective, several previous works regarding optical wireless systems for positioning [106,107,108]. They can be based on VLC systems, using different techniques [109,110,111], OCC systems [112,113,114], or even combining optical and ultrasound techniques [114]. Regarding welfare tourism, there have also been several research papers related to the use of OWC in hospitals or health facilities that can also be used in adapted hotels or residences [115]. The use of VLC services for visually impaired people has been studied in depth in [116], while specific medical applications are described in [117], and remote patient monitoring in [79] for those who should be followed by their medical services on their origin countries. Most of these applications can be combined with location/tracking systems to provide a complete service in specific, welfare service-oriented accommodations for impaired people, families, or senior citizens.

### 5.2. Indoor LiFi and LIoT Networks: Back- and Front-Office Services

Perhaps the use case that has received the most extensive attention, both from a commercial and scientific point of view, is high-speed indoor links based on lighting systems, in applications such as network management streaming [118,119] or VR [120]. These high-baud rate models, as was previously mentioned, are usually presented under the commercial name of LiFi©; they follow a long trajectory of previous developments, such as those based on infrared radiation [121], following or not the IrDA standard [122,123]. Companies such as pureLifi© [124], Phillips-Signify© [125], or Oledcomm© [126] have already presented different commercial solutions. Its final implementation in the market continues suffering problems mainly due to the need to integrate receiving devices in commercial equipment, to the consumption associated with emitting lamps [127,128,129], and synchronization problems when several transmitters coincide in the same room, working in a cellular architecture [130,131,132]. This type of solution may be of great interest in a tourist facility as it allows offering high-density services (video streaming, video conferences, gaming systems, and augmented or virtual reality) without overloading conventional radio links and with enhanced security compared to traditional wireless links.

Furthermore, for most of these applications, mobility problems can be minimized, as, in general, the user remains in a relatively fixed position observing a screen. It is also a complementary technology that does not intend to displace conventional radio systems. As RF and optics do not interfere with one another, they can work together in the same room or in adjoining rooms.

Regarding IoT, and beyond the location-based information used for SoCoMo solutions, one of its most important applications within hotel management is the so-called hyper-personalization. Hilton© and Marriott© have developed different models of a “connected room”, where users can control various services in their room (air conditioning and lighting) from a smart device, which can be their own smartphone, a control panel, or a tablet that can be provided by the hotel itself [133,134]. This instrument can allow guests not only to specify the ideal comfort conditions in their room through various regulation devices, but also to control their electricity or water consumption. Using this, a new business mode is opened for hotels to apply discounts or penalties in the rate for their sustainable or “green” behavior, depending on whether they have responsible consumption or above the expected standard. This type of control panel also allows to control the television or streaming channels, the aromas and to “personalize” the room, even greeting the tourist by his/her name, carry out parental control over the children’s television channels or a nutritional or allergenic verification on the menus. Light-IoT or LIoT applications, especially those that use image sensors as receivers [135,136], are very suitable to be part of this growing market.

Another potential use of the Internet of Things within the hotel industry is in the field of preventive maintenance, by providing hotel staff with up-to-date information on the operational status of different devices and appliances (either for systems directly used by customers or for those of the hotel facilities). This allows you to alert on any deterioration, or lower-than-expected performance. Repairs or replacements can be made before the device or apparatus completely collapses [137,138]. This benefit can be highly valuable on essential equipment for the hotel, allowing preventive repairs to be carried out before a significant breakdown or diagnoses with a view to obtain or maintain ISO certifications.

Another application area regards with room access, where check-in via electronic cards or apps has been a major improvement. Traditionally, hotels have relied on RFID access cards or, earlier, classic keys to unlock hotel rooms and control cleaning staff times, but IoT opens new possibilities. Virtual access cards can be sent directly to guests’ mobile phones, which can communicate (for example, via NFC) with the door lock, unlock the door and verify identity, (e.g., by biometric means), eliminating the need for a “key”. This app ca be sent in advance on arrival avoiding the wait at check-in time, or to all the smartphones of the components of a family unit or group of travelers. Lightkey© by Lightbee© [139] is the first VLC-based commercial door lock application for hotels to be implemented in a practical way. Other hotel companies such as Best Western©, Marriott©, and Wynn© [140] have been among the first to adopt voice-supported customer service, facilitating access for people with functional diversity and increasing the level of access security. This is an area that will probably expand substantially in the coming years, benefiting the companies and research groups that develop them. The ability to provide a superior customer experience and avoid contact with keyboards or surfaces has proven especially important during times of COVID. One example is voice-controlled room attendants that allow customers to order room service, reserve a table at the hotel restaurant, or book spa sessions simply by talking to a device in their room.

Awareness of the future potential of these systems by hotel owners can certainly increase investment and help automate processes, improve the customer experience, and help hotel companies save money on energy and maintenance costs. On the other hand, it is imperative to adequately ensure security of the “connected hotel”, as usually IoT systems are not adequately protected, they are vulnerable to undesired access, ransomware, and piracy. This is a risk to consider when developing smart hotels, and traditional wireless RF networks present greater security risks because they are less robust against malicious or simply careless users. Security issues appear to be much less challenging in VLC or OCC systems, something to be considered when measuring their market access potential as a way to reduce potential risks [141].

### 5.3. Underwater Optical Communications

There are many infrastructures in a hotel or tourist establishment that may require underwater communication or sensor systems. There are submerged hotels or water parks that offer attractions or panoramic views of marine fauna with facilities that need to be monitored [142,143], but most hotels have swimming pools, or spas are near beach or lake areas. There are also increasingly more proposals to extend IoT networks’ functionalities to underwater systems, for which there are different communication alternatives based on acoustic, RF, or optical systems [144]. Radio waves suffer from a high attenuation in water [145] and, at low frequencies, require large antennas. Underwater sound communications are influenced by propagation delay, path loss, multi-path fading, high bit error rate (from 10-5 to 10-2), and limited bandwidth, depending on range and frequency [146]. Besides, acoustic waves could distress swimmers. Therefore, acoustic communication is not the best solution underwater with applications that need high data rates and real-time operation [147]. Although they have a shorter range than acoustic ones, optical networks based on visible radiation offer higher transmission speeds and lower latency, and can be implemented with systems of lower consumption, size, and complexity than their radio frequency counterparts [148,149].

One of the possibilities opened by wireless optical systems is to interconnect scuba divers during their activity [150], either to receive instructions or assistance the training period or to provide guidance services in the case of guided excursions, e.g., when visiting a wreck, a coral reef, or an archaeological site. It also provides an additional safety level for novice divers as it can also incorporate optical emitters to monitor activity (e.g., oxygen level). Compared to other alternatives, such as acoustic systems, optical links reduce their impact on marine fauna and lower consumption and latency. Various developments have been made of systems for head-to-head communication or submerged sensors, for short distances and moderate transmission speed, such as voice communication for real-time swimmers’ feedback. Most of the proposals are based on blue-green wavelength, which has minimum attenuation for absorption and scattering. These systems generally offer short-range communication over 1 m between sensors mounted at the swimmer’s wrist or head. The link needs to communicate both in line-of-sight (LOS) or non-line-of-sight (NLOS) conditions and even deal with air bubbles generated by swimmers, which causes additional link attenuation, and ambient or artificial light sources. Other effects as turbulence can be neglected due to the short-range and baud rate required [151,152,153,154,155], unless other real-time video transmission systems have been presented [156].

## 6. Tourist Area Networks

The last level of definition corresponds to the systems that serve the traveler, which we have called Tourist Area Networks (TAN). Since the 19th century, when the railroad turned travelers into projectiles being shot through the landscape, they began to lose the sensory immersion, and landscapes became something perceived as distant, evanescent panoramic views. These TAN devices try to recover the feeling of being not a mere spectator but a protagonist of the trip. They are based on the concept of Wireless Personal Area Network (WPAN), which are those designed to cover distances up to a maximum of 10 m. These are user-oriented systems (human-centric applications), designed for medium–low transmission speeds and battery-powered, where power consumption to maximize their batteries’ lives is a significant design concern. The notion of travel brought by smartphones and other devices allows travelers to access and distribute information through the mobile communication network the Internet and allow careful examination and better perception in travel.

### 6.1. Wearable Systems

“Wearable” systems can be defined mobile electronic devices that can be integrated into the user’s attire, either in their clothing or in an accessory (glasses, caps, sweatshirts, etc.). Unlike conventional mobile systems, they are accessed in minimizing interference with user activities. These systems can also be used to model and recognize user activity or be context sensitivity to monitor our environment. Wearable concept cover from micro-sensors embedded in textiles, to consumer electronics embedded in running wristbands, glasses, and smart watches, or even belts with processors that communicate with a screen on a helmet or on the wrist. This concept of wearable computing is part of a broader framework of ubiquitous computing that seeks to improve interaction with our environment through smart electronic devices and has typically been used in health-related applications [157]. Continuous monitoring of certain constants can be a critical factor when travelers with chronic diseases (for example, coronary heart disease) may need urgent medical attention and an early diagnosis, but also for sports groups or teams doing winter stages in preparation for summer competitions when the climate in their origin countries prevents training.

There are several applications in which wearable devices with the optical transmission can be included as alternatives to conventional radio systems. An example would be using sportswear emitters to monitor physical activity during training on a treadmill, a stress test, or a running track [158]. The main advantage of using the smartphone camera as a receiver is comfort compared to wired systems and privacy related to wireless alternatives such as Bluetooth©, not only due to the greater directivity of the optical signal, but also because it can be encoded using color-space-based modulations or spatial division, further complicating its deciphering. Maintaining privacy is a relevant factor when what is being sent is health-related data as it can imply possible increases in insurance premiums, the non-renewal of a contract. It can also be used as an automatic recognition system for tracking routines aimed at children or people with reduced capacities in hotels or leisure areas. In general, OLED systems embedded in clothing are considered as emitters, such as those already on the market, and that does not have to emit a visible light pattern (in these cases, since they are short distances, IR systems can be used low power in the 800–900 nm band that a conventional silicon CMOS camera can perfectly capture).

Wearable systems are usually based on addressable LEDs, either in strips or on sewable PCBs, such as Neopixel or similar products, used in fashion or jewelry designs [159]. These strips can be used to make bracelets similar to the plastic or RF-based used in many hotels to enable access to the host reservation services (spa, gym, or food services). Nonetheless, they can now become active emitters for transmitting signals to OCC receivers, opening a complete, new range of additional services (for example, room key payment or location-based services [160,161]). Moreover, the Korean Institute of Advanced Science and Technology (KAIST) has created OLED fibers that can be woven into a fabric that can illuminate as individual dots to create a light source (e.g., in a cap if we are running by night) or addressable pixels, making the fabric behave like a complete LED display itself. This process does not require high temperatures in its manufacture so that almost the entire range of everyday textile fibers can be used as a substrate. The results are long-lasting and the fabric’s behavior as lighting elements is not affected under deformation, making them suitable garments [162,163].

Another wearable system family is head-mounted displays (HMD), both for Virtual or Augmented reality (e.g., in a theme park or museum). Head-to-head communication is needed to share the experience and implies communication between two fronts seated or standing at a considerable distance while using a head-mounted display. Almost all HMD device comprises a different camera to detect obstacles capturing photos and recording videos. OCC-based head-to-head communication also needs integrating an LED (usually IR LED for avoiding light interference) in each HMD device for positioning, mutual data exchange, or to update other necessary information. As only moderate baud rate values are needed, OCC technology offers non-interference communication, better SNR value and avoidance of pointing, and complex processing of the transmitted signal compared with VLC or RF connections [164].

### 6.2. OCC as an App

As already mentioned, the possibility of using OCC systems through devices (screen, camera, LED indicators), embedded in a smartphone offers a new market for VLC systems, as it will allow using OCC as an App and the integration of additional features in these devices. These services can offer great possibilities in the sector, as they can be configured as an access system (virtual key) to a hotel room or other services within it (Spa, restaurants). One of the significant interest areas can be payment services, either with a generic credit card or an ad hoc card (for example, in a theme park or a vacation resort) from the cell phone. These operations are currently based on NFC connections, but OCC offers additional levels of data security. In this case, a POS could be designed with an integrated camera and using the mobile screen as a transmitter of the customer data.

Nevertheless, smartphone-based real-time processing is a challenging issue. It has camera limitations, various operating systems (Android, Windows, or iOS), making that the same OCC application cannot be used in all types of devices different a lower processing strength compared with a regular laptop. However, soon, this technology could contribute to the upcoming IoT paradigms and the requirements of massive user connectivity. Among these operating systems, Android has the highest number of users worldwide; thus, it has an excellent prospect for creating applications in this area. Furthermore, image sensors do not have identical characteristics, as their imaging quality, sampling rate, and behavior vary for different smartphones. Android versions from camera2 API support manually controlled exposure, focus, and raw capture [165]. Moreover, the new CameraX is designed as a wrapper for solving device-specific compatibility issues. The higher camera sampling rates, the higher throughput can be offered for the OCC system, but these high sampling rate cameras are much costlier than those with low sampling rates [166]. $R_{f}$ frame rate should be fixed at a certain value to implement OCC using a smartphone camera. For a given frame rate, $R_{f}^{-1}$ is the time limit for each frame in real-time but, as all smartphone cameras are rolling shutter type [78], the processing lower time limit for an OCC App would be ${\left(N\cdot R_{f}\right)}^{-1}$, while the need of avoiding visual flickering will limit the higher processing timer.

Some specific modulation schemes have been proposed for this concrete application, as Undersampled Phase-Shift On-OffF Keying (UPSOOK) [9], Orthogonal Frequency Division Multiplexing (OFDM) [167], or combining Artificial Neural Networks (ANN) [168], while using managing data from multiple LEDs simultaneously, usually using spatial division or even wavelength division [169], can increase the baud rate, unless this last solution limits the maximum communication distance. In any case, many of the services that these applications must support, as could be location or guidance within a facility or the exchange of tokens between mobiles, do not require a large volume of data or a high baud rate.

This new integration opens the path to several new, information-oriented services provided by smartphone-based OCC applications [170]. The previously mentioned guidance services based on location routines can be based on image analysis (e.g., related to a set of pre-defined light beacons) [171,172]. Those systems can be integrated into the location or augmented reality systems for museums [173,174], considering not only that it is easy to get lost in a museum, especially if its floor plan is complicated, but circulation and attention patterns offer a view of visitors’ cultural and leisure experience and interests [175]. Providing solutions that help visitors orient themselves physically and cognitively seems particularly important for cultural institutions to fulfill their educational mission. It can also generate “heat maps” of interests, segmented by tourist characteristics, and even prevent crowds in particular masterpieces.

Smartphone applications can be used, combined or not with wearable devices, in some sports activities mostly related to tourism as golf, cycling, or city races (more than 100,000 people visited New York for the Marathon in 2019 pre-COVID age). Golf-related activities are a business over 100 Billion USD (in 2019), and there is a growing interest in augmented reality, positioning, or predictive apps for golf courses [176], that can also OCC for acquiring information from dynamic information panels. Companies as Zepp Golf© offer an IoT-powered smart coaching system, where a small sensor attached to the golfer’s glove measures and analyzes their golf swing to identify areas where the player can improve, such as how to adjust their club speed, club plane, tempo, or backswing length. The chip instantly sends customized feedback through a mobile app, offering training programs and video tutorials tailored to the player’s ability [177]. Alternatively, Arrcos© offers a set of connected golf club sensors (paired to a mobile app) that lets consumers track the distances reached for each club, ideally helping golfers improve their drives and overall score [178]. These systems have also been adapted for other sports like tennis or basketball and will other wearable sensors and combined with OCC readers.

The capability of extracting background information from commercial information panels, directly available or through steganography techniques, is another relevant feature of an image sensor-based receiver. It can be used as an improved QR code for personalized information, e.g., in an airport where the panel shows general flight information. However, a singular user can obtain specific information about his connections, luggage, or any other incident just pointing his flight with his smartphone camera.

## 7. The Future Evolution of the OWC-Based Services in the Post-COVID Tourism

The COVID crisis has meant a breaking point in the tourism industry, focusing its efforts on planning its recovery for the post-vaccines period. Even when the disease becomes chronic, clients themselves will demand a higher level of control and prevention than in the pre-COVID stage, so it will be necessary to install supplementary systems and services. Marketers believe that, from now on, travelers will modify some of their behavior patterns in their movements in the so-called “new normal”. Some major trends are pointed out in the post-COVID-19 scenario:

- Travelers, and the sector as a whole, will have to learn to live with the new social practices and the new protocols derived from the health crisis.
- Safe-guaranteed destinations will be prioritized; transparency and communication to project these destinations’ health reality will be crucial.
- More flexible reservations without cancellation fees will be imposed.
- Travelers will seek excellence in safe protection, and health insurance will play a more significant role.
- Proximity tourism (cultural, gastronomic) and non-mass experiences will be prioritized.
- Young people will probably be the first to return to travel. This fact will focus on main destinations offers.

For these reasons, a new communication model that highlights all the benefits of smart destinations will be needed: governance, good communications, safety and security, and health system, especially in the case of being one of the most infected (thus, in the road of becoming more immune). Quantitative and qualitative improvements in connectivity, sensorization, information systems, or online marketing will be required.

A recent study has presented the “Safe Destination” matrix [179], which offers a selection of technological solutions to respond to the different challenges that tourist destinations must face. This list of solutions is deployed on a matrix of two axes composed of public management spaces related to tourism and the challenges a municipality must address to provide guarantees to its tourists (Table 2). The measures are aimed to reduce contact (both person-person or person-object), the control of social distancing, or the early detection of symptoms related to COVID. Solutions must also estimate the tourist’s perception of the destination and create direct real-time communication channels to keep visitors informed of any incident. Specific solutions for closed enclosures include decontamination systems or a Health Care module, integrating a digital information panel with a contactless disinfectant gel or dispenser, and the option to measure the user’s temperature. Open, outdoor areas will also be controlled to guarantee compliance with social distancing through capacity control cameras, drones, and heat maps based on Wi-Fi networks, among other solutions.

Many of these solutions can also be implemented using wireless optical systems. Thermal cameras can be used as temperature gauges and as OCC receivers, while information panels or Urban Furniture as an Information Point (UFIP) can be used as OCC emitters for smartphones, either directly or using steganography techniques. The location or tracking techniques based on VLC or OCC will allow positioning people with special needs (senior citizens and children), while the Apps with OCC can be used in contactless systems to make payments, appointments, on the check-in process of a tourist on his arrival to the hotel or included in smart parking or smart market apps. These routines could be used as complementary to others, such as QR codes, RFID, or NFC systems in COVID tracking routines to identify users in a given space or their contacts.

Furthermore, in commercial premises lighting can complement LED-based signaling systems on the ground (or from the ceiling) to dynamically mark distances between clients or manage dynamic entry–exit routes to avoid crowds. There are also several works regarding the use of UV systems for surface disinfection [180], as it has been proven that far-UVC light (207–222 nm) efficiently kills pathogens potentially without harm to exposed human tissues [181,182]. The use of UV systems for communications has been usually neglected due to health and cost considerations, but now the recent and rapid developments in deep ultraviolet LEDs, solar blind ultraviolet filters, and detectors, suitable to be used for communications and sensing in either line-of-sight or non-line-of-sight channel conditions has prompted the interest of using UV signals in OWC, in [183] there where proposed some potential communication systems but limited in power. They can be used as data sources for sensor networks or positioning the robot itself to be sure all areas have effectively cleaned [184]. Detection systems for this wavelength has been proposed in [185], as well as the use of hyperspectral cameras for UV-based OOC systems [186].

## 8. Conclusions

This paper has presented various technological solutions based on OWC for different application environments in the tourism industry, which, together with other classic solutions, both wired and wireless based on RF systems, can provide connectivity for new smart tourist destinations. Solutions based on image sensors’s use as receivers seem especially promising as they allow progress in the commercialization of optical systems by allowing them to be integrated into a smartphone as an app. Incorporating these technologies will be carried out to modernize the tourism industry, which will be needed after the current health crisis, which will cause a significant increase in the demand for data to feed the behavioral analysis systems, part of the paradigm IoB. These assumptions make IoT technologies an evident bet in smart tourist destinations and hospitality and leisure complexes. These results will be fed CRM systems, by these results, so the availability of unregulated means of access, safe for the data and the user, and free from interference with the installed systems will undoubtedly become a desirable technology for these markets.

It is hard to provide a detailed cost analysis derived from the implementation of the different OWC systems, as we are comparing a mature, well-established technology with thousands of providers with an emerging one. Nevertheless, in the case of OCC, as the trend would be to work as a mere app for smartphones, and once the control of these systems is incorporated into the APIs, its cost will be minimal and therefore they will coexist with bluetooth applications, even with a lower cost of energy consumption, something significant since it is dependent of a limited source such as phone batteries. FSO systems are already commercial and they coexist with radio-frequency radio links. The cost analysis should be then not only limited to its installation and maintenance value (higher than in a conventional radio link), but also reflect other parameters such as the nonexistence of license costs for the use of the spectrum and greater security in the link. In addition, there is a market opportunity given by its better operation in areas of severe rains, which affect the performance of RF systems much more than the FSO, such as resorts or facilities in areas of humid forest. Conventional VLC has cost and difficulty of implementation greater than systems with an almost ubiquitous presence such as WiFi or LTE/5G, so its potential application is getting restricted to those sectors not covered (or with saturation problems) by conventional RF systems, such as location systems, devices embedded in clothing or underwater communications networks, or in those where the presence of RF systems can be discouraged, as would be the case of heritage buildings or museums, where the installation of simple lighting systems does not have an appreciable visual impact on the image of the building.

Furthermore, during the intermediate period between the recovery of normality, brought by the progressive introduction of vaccines, and the full recovery of the sector (which some market estimations place not before 2023), hotels and destinations should make an additional effort to equip themselves with systems that provide customers with a certain degree of security. Some of these systems are contactless systems, contact tracing or customer tracking technologies, thermal cameras for the early detection of possible infections, or artificial intelligence systems to minimize interactions between hotel staff and customers. Many of these applications can be based on OWC systems, or require cameras that can be used twice, creating new VLC and OCC business opportunities.

The possibility of incorporating data reception routines to smartphone cameras brings the opportunity of converting visible light communication systems into an additional app into a mobile system. This opens new markets such as sensorization, home automation, payment, and tracking and location applications, all of them of interest in the tourist environment. Information panels, lighting systems or even the screens of mobile phones can also become transmitters. In this way, optical systems can make the final leap to become a realistic commercial complementary service to radio frequency-based systems.

## Figures and Tables

**Table 1:** Some significant initiatives in the use of ICT for Tourist destinations.

Project	Definition	Technologies	(E/I)	Scenario
5G Interactive City, Barcelona	Allows visitors to discover the interior of monuments, shops, and entities; see related content; or buy virtually thanks to Mixed Reality glasses connected with 5G technology.	Magic Leap glasses, Augmented Reality, and 5G networks	E/I	SD, TSF, ST
Big data solution for tourism in Alcalá de Henares	From smartphone data, studies the real behavior of hikers and tourists, nationals and foreigners, in different geographical areas corresponding to Alcalá de Henares municipality.	Big Data, Cellular networks, LUCA Tourism Service (Telefonica Data Unit), and Internet of Behaviour.	E/I	SD
MAGICBAND	Uses a bracelet, including RFID technology and multiple readers to control access to hotels and resorts, fast pass services, and track users’ movements and preferences.	Wearables, Internet of Things, Internet of Behaviour, RFID	E/I	TSF, ST
Blockchain in the tourism sector (TUI Business Group)	TUI Business Group uses blockchain for the management of all its reservations and internal processes. Implies cost savings and potential disruption to the sector based on disintermediation.	Blockchain	E	TSF
Tech Room (Melia Hotel International)	Future hotel room, customizable to fit clients’ preferences and demands. The room will interact with users through electronic systems and AI.	Multiple digital technologies (digital lock and a smart key, cleaning robot, Internet of Things, sensors, etc.)	E/I	TSF

**Table 2:** Safe destination matrix developed from the work in [178].

		Social Distance	Contact-Less	Symptom Detection	Intelligence	Real Time
Open Areas	Beach	Capacity Control Cameras				Destination app to check capacity on the beach
	Golf resort		Healthcare modules			UFIP with real time information
	Camping	Access Control		Thermal cameras	Big Data Semantic Social Network Monitoring Segmented Tourism Reports	
	Nature	Thermal maps: drones and thermal cameras	IoT Traffic Lights			
Closed enclosures	Tourism office	People-counting sensors	Decontamination system			Chatbot call centers
	Hosting	App assigns restaurant or Spa turns	Taps and electrical switches Online check-in/check-out			
	Museums	Beacons and information panels	Online access Management			Non-touch screens with QR and voice controlled
	Local markets	Floor illumination signaling	Marketplace Apps			SMS platform
	Parking	Smart Parking IoT	Gel Dispensers	Temperature or face mask detection using cameras	Traffic control through Big Data	Visual panels
Mobility	Public transport	On-board WiFi	City cards, NFC, or SMS payment		Access control database	Voice overs with security warnings Information panels
	Stations, ports, and airports	Access Control and ID				
Health	Health centers	Appointment management	Video-conference system App assigns turns	COVID Apps		Web connected with smart destination platform
	Senior travellers	Location and tracking				
